# Low-Temperature Selective Catalytic Reduction DeNO_X_ and Regeneration of Mn–Cu Catalyst Supported by Activated Coke

**DOI:** 10.3390/ma14205958

**Published:** 2021-10-11

**Authors:** Xusheng Ren, Zhiliang Ou, Bang Wu

**Affiliations:** 1 Key Laboratory of Low-Grade Energy Utilization Technologies and Systems of Ministry of Education, School of Energy and Power Engineering, Chongqing University, Chongqing 400044, China; renxusheng33@163.com; 2 State Key Laboratory of Coal Combustion, School of Energy and Power Engineering, Huazhong University of Science and Technology, Wuhan 430074, China

**Keywords:** activated coke, low-temperature NH_3_-SCR, sulfur poisoning, regeneration

## Abstract

The activated coke is a promising support for catalysts, and it is important to study the performance of the activated coke catalyst on the removal of NOx. In the current research, a series of the activated coke-supported Mn–Cu catalysts are prepared by the incipient wetness impregnation method. The effects of the molar ration of Mn/Cu, the content of Mn–Cu, the calcination temperature, and reaction space velocity on NO conversion are investigated, and it was found that the 8 wt.% Mn_0.7_Cu_0.3_/AC had the best catalytic activity when the calcination temperature was 200 °C. The existence of SO_2_ caused the catalyst to deactivate, but the activity of the poisoning catalyst could be recovered by different regeneration methods. To uncover the underlying mechanism, BET, XPS, XRD, SEM and FTIR characterizations were performed. These results suggested that the specific surface area and total pore volume of the poisoning catalyst are recovered and the sulfite and sulfate on the surface of the poisoning catalysts are removed after water washing regeneration. More importantly, the water washing regeneration returns the value of Mn^3+^/Mn^4+^, Cu^2+^/Cu^+^, and O_α_/O_β_, related to the activity, basically back to the level of the fresh catalyst. Thus, the effect of water washing regeneration is better than thermal regeneration. These results could provide some helpful information for the design and development of the SCR catalysts.

## 1. Introduction

Nitrogen oxides (NO_X_) are the main pollutants in the atmosphere, and these pollutants can cause chemical smog and acid rain, which further result in significant damage to the ecological environment and human health [1,2]. The selective catalytic reduction (SCR) of NO_X_ with NH_3_ (NH_3_-SCR) technology has long been a mature and extensively used method for controlling NO_X_ emissions [3,4]. However, the commercial vanadium–tungsten–titanium catalysts in the process are expensive due to the use of precious metals as raw materials, and the vanadium species is also a toxicant; thus, they are unfit to be applied in long-term industrial enterprises. More importantly, the optimum temperature ranges for the use of the vanadium–tungsten–titanium catalysts are 300~400 °C, which does not match with the low-temperature selective catalytic reduction process. In recent years, many scholars have attempted to develop efficient, cheap and low-temperature SCR catalysts [5,6,7,8,9]; in particular, the activated carbon attracted extensive attention. As a carbon-based porous medium, activated coke has similar structural characteristics to the activated carbon, and its chemical stability is basically the same as the activated carbon. Though the physical adsorption performance, chemical adsorption performance and catalytic activity are slightly lower in activated coke than in activated carbon, but the former has a lower price, high mechanical strength, and strong adsorption properties. Therefore, activated coke is highly suitable as a carrier for catalysts, and it has very important significance in studying the performance of the activated coke catalyst on the removal of NOx.

In recent years, many scholars have conducted considerable research on carbon-based SCR catalysts. They found that carbon-based materials are beneficial for adsorbing NO and NH_3_, and they improve the dispersibility of the surface active components and promote the generation of lattice oxygen, which leads to the formation of amino compounds and further promotes the catalytic reaction cycle [7]. In addition, Cu^1+/2+^ and Mn^2+/3+/4+^ also have significant catalytic effects on SCR reaction; thus, the extensively distributed Cu and Mn oxides are considered to be promising low-temperature SCR materials [6]. The carbon-based materials-supported Mn catalyst in the NH_3_-SCR reaction can exceed 90% of the conversion rate below 200 °C, while the supported Cu catalyst could increase the surface active site and further promote the SCR reaction [7,10]. Moreover, the modification of Mn can further improve the denitrification activity [11], and Cu can enhance the N_2_ selectivity of the catalyst [12]. However, there are few reports focusing on the activated coke-supported Mn–Cu catalysts for SCR reaction, and the reaction mechanism of Mn–Cu catalyst in the NH_3_-SCR reaction is still unclear [1,7,13]. In addition, the low-temperature SCR deNO_X_ efficiency and deactivation regeneration technology of the Mn–Cu/AC catalyst are also need to be investigated in order to develop the catalyst.

In the present work, a series of Mn–Cu/AC catalysts were prepared by the incipient wetness impregnation method. A detailed experimental study was conducted on the tail flue of an industrial boiler to determine the optimum molar ratio of Mn/Cu, the content of the Mn–Cu, and the effects of the reaction temperature and space velocity on the NO_X_ conversion rate are uncovered. Moreover, the physical structure and chemical properties of the catalysts were studied through XRD, BET, XPS, SEM and FTIR characterization to understand the underlying mechanisms. Furthermore, the catalyst deactivation mechanism was determined by simulating catalyst sulfur poisoning, and the effects of different regeneration methods on the NO_X_ conversion rate of the catalysts were also investigated.

## 2. Materials and Methods

### 2.1. Catalyst Preparation

The activated coke used in the current experiment was 4 mm-diameter columnar activated coke particles, provided by Zhulin Activated Carbon Co., Ltd. (Zhengzhou, China) The catalyst was prepared by the incipient wetness impregnation method. Firstly, the purchased active coke was ground and screened, and the 0.1~0.5 mm activated coke particles were left for further use. Secondly, the left activated coke was dried in a blast drying oven (YIHENG DHG-9070(A), Shanghai, China) at 110 °C for 10 h. Then, the activated coke was soaked in diluted nitric acid (20%) (Xingguang Chemicals, Chongqing, China) for 10 h, after being washed three times with deionized water, and dried in a blast oven at 110 °C for 10 h to completely remove the water. Thirdly, impregnation was performed according to the molar ratios of Mn/Cu, and the manganese nitrate and copper nitrate (Analytical Reagent, obtained from Aladdin Chemicals) (Shanghai, China) were dissolved in distilled water and completely mixed. Then, a certain amount of activated coke was added into the solution and it was shaken with ultrasonic for 30 min. The prepared samples were dried in the drying furnace for 10 h at 110 °C. The molar ratios were Mn_0.9_Cu_0.1_, Mn_0.7_Cu_0.3_, Mn_0.5_Cu_0.5_, Mn_0.3_Cu_0.7_, and Mn_0.1_Cu_0.9_, respectively. The contents of Mn–Cu in the catalysts were 6 wt.%, 7 wt.%, 8 wt.%, 9 wt.%, 10 wt.%, respectively. Finally, the samples were calcined in a tube furnace (CHENGYUE CY-O1200-50I-T-V, Zhengzhou, China) under the protection of nitrogen for 4 h; the calcination temperatures were 200 °C, 250 °C, 300 °C, 350 °C, 400 °C, 450 °C and 500 °C respectively.

### 2.2. Catalyst Characterization

In this work, the nitrogen adsorption/desorption isotherm was measured at 77 K before N_2_ adsorption using a fully automated multistation surface and pore size analyzer (BET, Quadrasorb 2MP, Quantachrome, Boynton Beach, FL, USA). Furthermore, the pore size distribution and specific surface area of the catalyst were calculated. All samples were degassed at 160 °C for 8 h, and the specific surface area was calculated through the Brunauer–Emmett–Teller (BET) method because it is suitable for samples with typical Type IV adsorption/desorption isotherms. The pore size was measured through the Horvath–Kawazoe method, and the mesopores were measured through the Barret–Joyner–Halenda method. The catalyst powder was characterized by X-ray diffraction (XRD, PANalytic X’pert Pro, Spectris Pte. Ltd., Almelo, The Netherlands). The scanning rate was 15°/min, and the scanning range was 10° ≤ 2θ ≤ 90°. The surface morphology of the catalyst under monochromatic Al-Kα radiation (15 kV, 150 W) was measured by X-ray photoelectron spectroscopy (XPS, ESCALAB 250Xi, Thermofisher, Waltham, MA, USA). The instrument base pressure was 10^−9^ Torr. High-resolution spectra were acquired using an analysis area of 500 μm × 500 μm, and transmission energy of 10 eV. The surface morphology of the catalyst was characterized by field emission scanning electron microscopy (SEM, SU8020, Hitachi, Japan). The catalyst was characterized by Fourier transform infrared spectroscopy (FTIR, Nicolet iS50, Thermofisher, Waltham, MA, USA) to analyze the functional groups contained in the catalyst.

### 2.3. Activity Test

**Figure 1:**
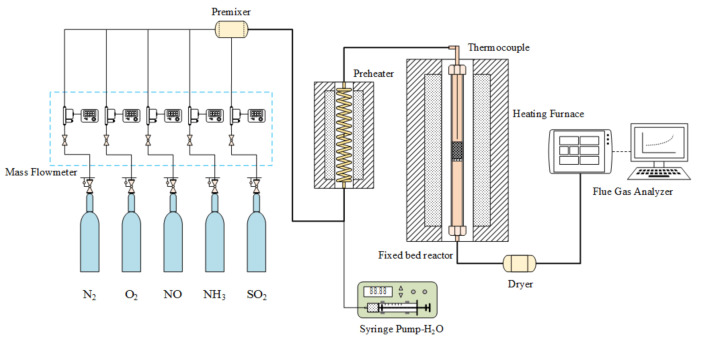
Fixed bed reactor.

The experiment was conducted in a fixed bed reactor using a stainless steel tube as a reaction tube (20 mm in diameter) and a 12.9 g catalyst was placed in steel tube in each experiment. The simulated flue gas composition was 530 ppm NO, 530 ppm NH_3_, and 6 vol.% O_2_, and the remaining carrier gas was N_2_. The total flow of the experimental gas was 2.2 L/min. The experimental temperature was controlled at five temperature points (i.e., 120 °C, 140 °C, 160 °C, 180 °C, and 200 °C). After running the experiment at the required temperature for 2.5 h to stabilize, the catalytic reduction activity test was performed under steady-state flow, and the outlet concentration of NO was measured online by a flue gas analyzer (Ecom J2KN, ECOM, Morrisville, NC, USA). A schematic of the experimental apparatus is illustrated in Figure 1. The percent conversion of NO is defined as follows:

(1) $$\eta_{{\mathrm{NO}}_{x}}=\frac{C_{{\mathrm{NO}}_{x}\left(\mathrm{inlet}\right)}-C_{{\mathrm{NO}}_{x}\left(\mathrm{outlet}\right)}}{C_{{\mathrm{NO}}_{x}\left(\mathrm{inlet}\right)}}\times 100\%$$

## 3. Results and Analysis

### 3.1. The Effects of Various Factors on Catalyst Activity of the Mn–Cu/AC Catalyst

**Figure 2:**
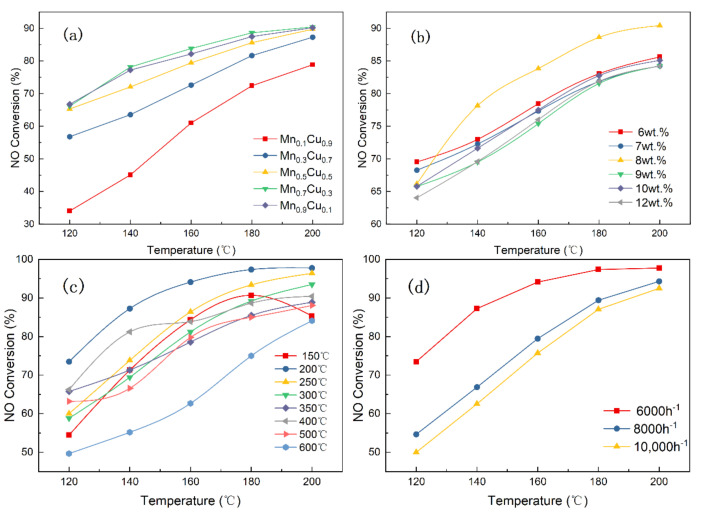
Catalyst activity of (**a**) different molar ratios, (**b**) different mass fraction, (**c**) different calcination temperatures, and (**d**) different space velocities (reaction conditions: NO = NH_3_ = 530 ppm, O_2_ = 6 vol.%).

The effects of the Mn/Cu molar ratios, the contents of Mn–Cu, calcination temperature and space velocity on catalyst activity are investigated in the current section, and the results are displayed in Figure 2.

Figure 2a demonstrates the effects of different molar ratios of Mn/Cu on the NO conversion when the content of Mn-Cn is 8 wt.% under the calcination temperature of 400 °C and a space velocity of 6000 h^−1^. As displayed in Figure 2a, the NO conversion shows a trend of gradual increase with the increase of the molar ratio of Mn/Cu, which is because the manganese is the main active component, and the deNO_X_ effect of the catalyst is significantly improved with the increase in manganese content. When the molar ratio of manganese to copper is 1:9, the NO conversion rate is the lowest in all catalysts, and the NO conversion rate is 78.83% at 200 °C. The Mn_0.7_Cu_0.3_ catalysts had the highest activity, which is equivalent to the activity of the Mn_0.9_Cu_0.1_ catalyst. These results suggested that the promotion effect of Mn on NO conversion is slight when the molar ration of Mn/Cu is more than 7/3. Figure 2b displays the effects of the contents of Mn–Cu on the NO conversion when the molar ratio of Mn/Cu is 7:3 based on the obtained results. It can be seen that the NO conversion shows a trend of first increasing and then decreasing with the increase of the content of Mn–Cu in catalyst. When the content of the Mn–Cu was 8 wt.%, the NO conversion rate of the catalyst was the highest and it reached 90.42%. Interestingly, the catalytic activity for the 8 wt.% catalyst is not optimal at 120 °C, but it shows better catalytic performance than other catalysts at above 120 °C. Hence, the optimal content of Mn–Cu in the catalyst is 8 wt.%.

The crystal structure of the supported metal oxide will change at different calcination temperatures, which could affect the catalytic performance of the catalyst. Hence, the effects of calcination temperatures on the NO conversion is investigated when the content of Mn–Cu in the catalyst is optimal. As displayed in the Figure 2c, when the calcination temperature is 200 °C, the catalytic activity is the highest and the NO conversion rate is 97.77%. Figure 2d exhibits the effects of reaction space velocities on the 8 wt.% Mn_0.7_Cu_0.3_/AC catalyst. When the space velocity is 8000 h^−1^, the NO conversion is 94.31% at 200 ℃, and the NO conversion drops to 92.51% at 10,000 h^−1^. Obviously, the NO conversion rate of the catalyst decreases with the increase in the reaction space velocity. The reason for this may be that the time for the reactants staying in the reaction tube and the reaction time are shortened as the space velocity increases, thereby resulting in a decrease in NO conversion.

In conclusion, the 8 wt.% Mn_0.7_Cu_0.3_/AC catalyst has the highest catalytic performance after calcining at 200 °C.

### 3.2. The Effects of the Regeneration Strategies and the Number of Times on the Catalytic Performance of Mn–Cu/AC Catalyst

The performance of the catalysts is affected by various conditions in the real environments, especially SO_2_, which could make the catalyst poisoned and deactivated. To reuse catalysts and reduce costs, it is necessary to use regeneration methods to restore all or part of the catalytic activity of the used catalysts. Hence, the effects of different regeneration methods on the 8 wt.% Mn_0.7_Cu_0.3_/AC catalyst are studied in the current section.

**Figure 3:**
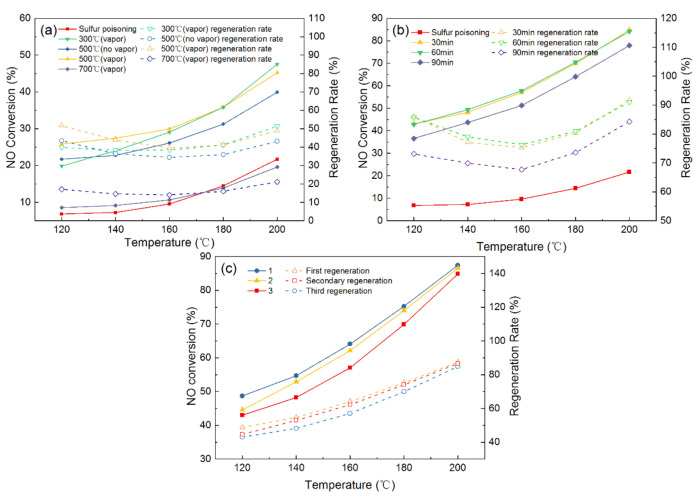
Catalyst activity and regeneration rate after (**a**) thermal regeneration at different temperatures, (**b**) regeneration at various washing times, and (**c**) regeneration frequency.

Before the regeneration experiment, the fresh catalyst is pretreated at 200 °C under the conditions of 300 ppm SO_2_, 6 vol.% O_2_, 6 vol.% H_2_O (g) to make the catalyst completely poisoned. The catalyst is deactivated and subsequently regenerated. Figure 3a illustrates the effects of different regeneration temperatures and the addition of water vapor on the sulfur poisoning catalysts. It can be seen that the catalytic activity decreased significantly after SO_2_ treatment and the NO conversion is only 21.68% at 200 °C, and the NO conversion of the used catalyst could reach 38.5% after thermal regeneration under the regeneration temperature of 500 °C. Moreover, the addition of water vapor during the thermal regeneration process could further improve the catalytic activity, and the NO conversion is 45.21% at same conditions. These results suggested that the addition of water vapor has positive effects on the regeneration of the poisoning catalyst; thus, the addition of water vapor is applied in the subsequent thermal regeneration experiments. For the different regeneration temperatures, the 300 °C regeneration temperatures had the highest NO conversion (47.56%) and the regeneration rate was 54.41% when the reaction temperature was 200 °C. The NO conversion and regeneration rate decrease as the regeneration temperatures increase. At the 700 °C regeneration temperature, the NO conversion and regeneration were 19.58% and 21.17%, respectively, which may be because the excessive temperature destroys the surface structure of the catalyst.

The water washing regeneration method is also studied and the detailed results are displayed in Figure 3b. Compared with the thermal regeneration, the NO conversion and regeneration rate of all the used catalysts by the water washing are significantly higher, and the NO conversion and regeneration rate are more than 70% and 80%, respectively, at the 200 °C reaction temperature. These results demonstrated that water washing regeneration has significant advantages in comparison with thermal regeneration. This may be because that sulfate impurities on the surface are removed by water washing and the active sites, contributing to SCR reaction, are recovered. Moreover, the NO conversion and regeneration rate are almost the same after the catalyst is washed in water for 30 min or 60 min. However, the NO conversion and regeneration rate is reduced after washing for 90 min, which is attributed to the fact that washing for a long time may remove part of the active component and lower the activity of the catalyst. Hence, the water washing regeneration method is a priority, and the optimal washing time is 30 min for the regeneration of the sulfur poisoning catalysts.

After obtaining the optimal regeneration methods, the effect of the number of times water washing regeneration is performed on the catalytic performance are further investigated, as displayed in Figure 3c. The NO conversion are 84.86%, 83.54%, 81.85%, respectively, after 1, 2, and 3 times of being water washed. These results suggested that the NO conversion of the regenerated catalyst has a slight decrease as the number of times of water washing regeneration increases. In general, the Mn–Cu/AC catalyst could retain higher catalytic activity after regenerating.

### 3.3. The Physicochemical Properties of the Mn–Cu/AC Catalyst

To understand the underlying mechanism of the regeneration process on catalytic performance, the physical structure and chemical properties of the catalysts were studied through BET, XPS, XRD, SEM and FTIR characterization.

Table 1 lists the specific surface, pore volumes, and pore diameters of different catalysts. The fresh catalyst has the largest specific surface area and pore volume, which is because that the pore structure of activated coke increased after nitric treatment. The specific surface area, total pore volume, and pore diameter of the catalyst after deNO_X_ reaction are reduced, while the specific surface area and total pore volume are the smallest after sulfur poisoning. SO_2_ forms sulfate and sulfite on the catalyst surface, thus the pore structure of catalyst changes. After the thermal regeneration, the specific surface area of the poisoning catalyst was recovered, but it was unapparent. It is worth noting that the effects of the water washing regeneration are remarkable, and the specific surface area, total pore volume, and pore diameter are restored to a state which is close to the catalyst after the deNO_X_ reaction. Hence, the water washing regeneration could better recover the catalytic activity of the poisoning catalyst.

**Figure 4:**
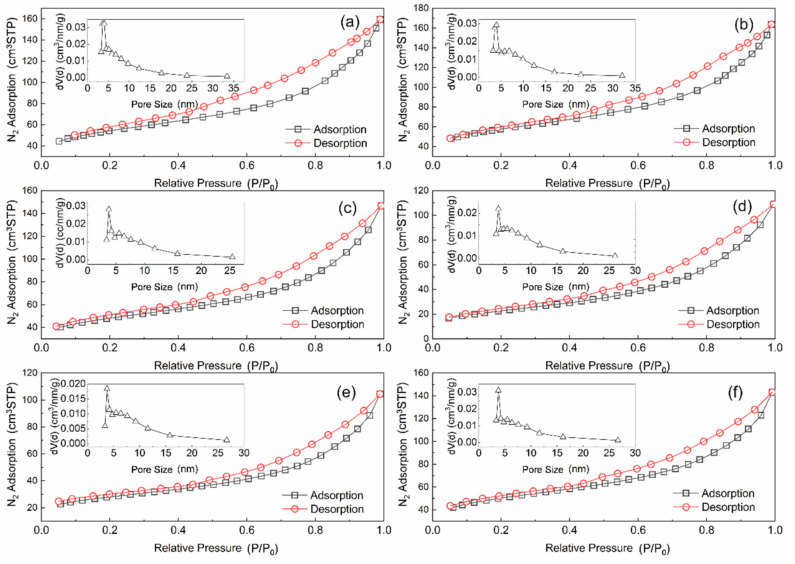
Nitrogen adsorption–desorption isotherm and pore size distribution of (**a**) AC, (**b**) fresh catalyst, (**c**) after deNO_X_, (**d**) sulfur poisoning, (**e**) thermal regeneration, and (**f**) rinsing regeneration.

Figure 4 depicts the N_2_ adsorption–desorption isotherm and pore size distribution of the different catalyst. All samples are Type IV (A) isotherms and have an H4 hysteresis curve. These results indicate that AC and Mn–Cu/AC are mesoporous carbon materials that produce narrow fracture pores [14]. The adsorption characteristics of mesoporous are the interaction of adsorbent–adsorbed substances and the interaction among molecules in a state of aggregation. After sulfur poisoning, the Mn–Cu/AC catalyst is still mesoporous carbon material. The adsorption amount in the low-pressure section is gradually increased. At this point, the substances are adsorbed on the inner surface of the mesoporous from a single layer to multiple layers and then agglomerated in the pores, accompanied by a hysteresis loop.

**Figure 5:**
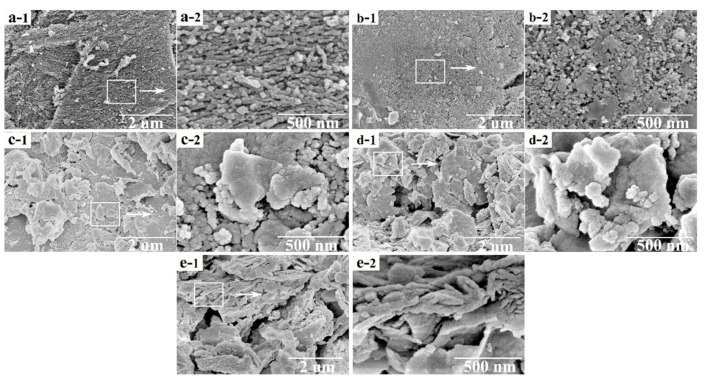
SEM surface topography of (**a**) AC, (**b**) fresh catalyst, (**c**) sulfur poisoning, (**d**) thermal regeneration, and (**e**) rinsing regeneration. Figure (a-1, b-1, c-1, d-1, e-1) shows the surface morphology of activated coke at 20,000 times magnification. Figure (a-2, b-2, c-2, d-2, e-2) shows the surface morphology of activated coke at 100,000 times magnification.

Figure 5 depicts an SEM image of the different catalysts. Figure 5a presents the surface morphology of the activated coke, whose surface is uneven and rough, and there are some impurities on the surface. Moreover, the surface is textured. Figure 5b exhibits the surface morphology of the fresh 8% Mn_0.7_–Cu_0.3_/AC catalyst calcined at 200 °C. The surface is relatively flat with traces of sintering, but there are few impurities, which is ascribed to the acidification of nitric acid. The treatment of nitric acid perhaps changes the pore structure of the activated coke and removes the impurities. In addition, the calcination process could make the surface morphology change. Notably, the supported metal oxide has a granular distribution. Figure 5c displays the surface topography at different magnifications after catalyst deactivation. As seen in the figure, massive condensate occurs on the surface. In comparison with the morphology of the fresh catalyst, the surface of the catalyst is no longer a uniform granular distribution but a large lump group, which may be because the adsorption of SO_2_ on the catalyst forms sulfate and sulfite, which then adheres to the catalyst surface. Figure 5d illustrates the surface morphology of the catalyst after thermal regeneration. The surface morphology of the catalyst is similar to that of the deactivated catalyst, and there are numerous bulk materials formed on the surface. These results suggested that the surface morphology has not been significantly improved and thermal regeneration has limited effects on the deactivated catalyst. Figure 5e demonstrates the surface morphology of the deactivated catalyst after water washing. The original block structure is broken and the condensation on the surface becomes smaller, which may be because sulfate that blocks the pores are removed and the surface can slightly recover after water washing. Therefore, the effect on the deactivated catalyst is significantly better after water washing regeneration than after the thermal regeneration method.

**Figure 6:**
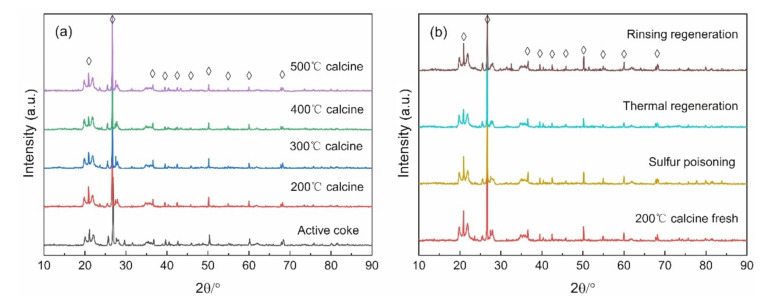
XRD pattern of (**a**) different calcination temperature catalysts, and (**b**) various regeneration mode catalysts.

Figure 6a displays the XRD pattern of the catalyst at different calcination temperatures, and Figure 6b presents the XRD spectrum of the fresh catalyst after thermal regeneration, water washing and sulfur poisoning. The characteristic peaks located at 20.88°, 21.62°, 36.54°, 39.44°, 42.43°, 45.82°, 50.13°, 54.85°, 59.99°, and 68.11°, belong to the SiO_2_ crystal phase (PDF # 99-0088), which may be an additive to the activated coke during the factory production process. There are no MnO_X_- and CuO_X_-related peaks detected. This result may be due to the low content of MnO_X_ and CuO_X_ or the uniform dispersion on the catalyst surface, and it is favorable to SCR reaction [15,16]. After Mn and Cu are doped and calcined at different temperatures, the peak intensity decreases with the increase of temperature, indicating that the high calcination temperature may make the Lewis site disappear and lower the catalytic activity. This can also explain the fact that the catalyst calcined at 200 °C had the highest catalytic activity in Section 3.1. After sulfur poisoning, the peak intensity decreased and recovered after regeneration, particularly after the water washing regeneration.

**Figure 7:**
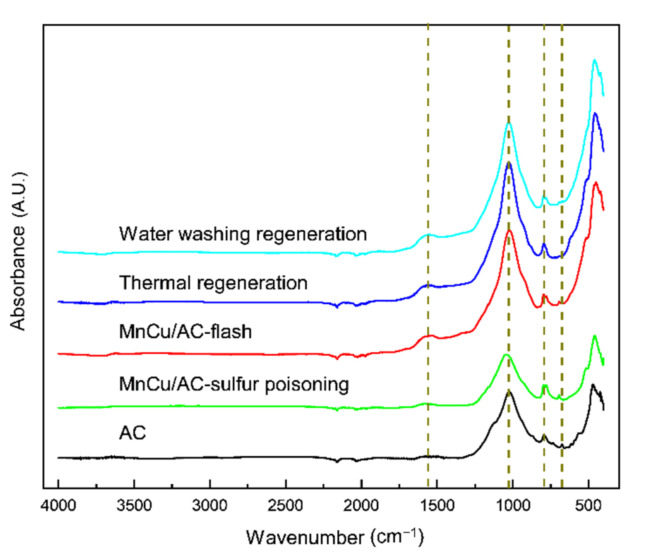
FTIR spectrum.

Figure 7 illustrates the FTIR spectrum of AC and different catalysts. The absorption peak at 1560.2 cm^−1^ generally belongs to the anti-symmetric stretching vibration of the COO of carboxylic acid and the in-plane bending vibration of the COH of alcohols. The band located at 1023–1042 cm^−1^ is attributed to stretching vibration of C-OH and C-O, symmetric stretching vibration of C-O-C, and antisymmetric stretching vibrations of C-O-C. The absorption peak at 794 cm^−1^ is attributed to the C-O-S symmetric stretching vibration [16,17]. The absorption peak at 695 cm^−1^, which is in the presence of the activated coke, the sulfur poisoning catalyst, and the fresh catalyst, is attributed to the C-S stretching vibration, while this peak is undetected in the catalysts after the thermal regeneration and water washing regeneration. These results suggested that the surface functional groups of the sulfur poisoning catalyst, located at the 1560.2 and 1023–1042 cm^−1^ positions, are destroyed. It is worth noting that the catalysts after thermal regeneration and water washing regeneration have similar strong absorption peaks, and the activated coke and the sulfur poisoning catalysts have similar weak absorption peaks. This is because the surface functional groups of the sulfur poisoning catalyst are destroyed and recovered after regeneration, which is similar to the fresh catalyst. In conclusion, the COH, COC, C-OH, and COO on the surface may have important effects on the activities of the catalyst, and the thermal regeneration and water washing regeneration may destroy the C-S bond of the AC surface; thus, the catalytic activity of the poisoning catalyst is recovered after regeneration.

**Figure 8:**
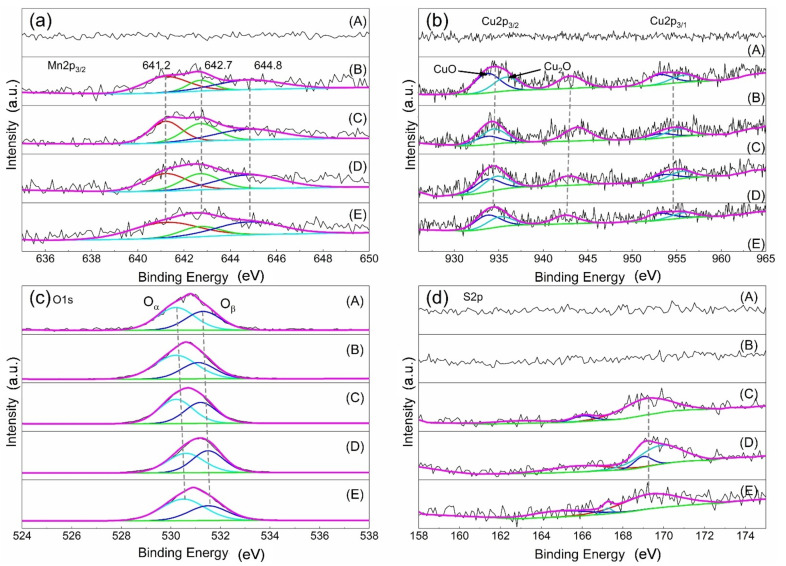
XPS spectrum of (**a**) Mn2p, (**b**) Cu2p, (**c**) O1s, (**d**) S2p. A, AC; B, fresh catalyst; C, sulfur poisoning; D, thermal regeneration; E, rinsing regeneration.

Figure 8 depicts the XPS spectra of Mn2p3/2, Cu2p, O1s, and S2p of the different catalysts, and XPS characterization results and surface element composition are displayed in Table 2. Except the AC, there are manganese species in the other samples. The peak at 641.2 eV is Mn^3+^, and the peaks at 642.7 eV and 644.8 eV are Mn^4+^, respectively [18,19,20]. The value of Mn^3+^/Mn^4+^ in the fresh catalyst is 2.63%, and it is 0.95% after sulfur poisoning. When the poisoning catalyst undergoes regeneration, the Mn3+/Mn4 rises; in particular, after water washing regeneration, it reaches 2.62%, similar to the fresh catalyst. These results suggested that the catalyst with high Mn3+/Mn4+ has high catalytic activity for SCR reaction, and the Mn^3+^/Mn^4+^ could be restored completely by the water washing regeneration method; thus, the activity of the poisoning is restored. Figure 8b demonstrates the XPS spectrum of Cu2p of different catalysts. The peak occurring at 934.1~935.8 eV is Cu+, and the peak appearing at 933.7~933.8 eV is Cu^2+^_,_ respectively. In addition, the peak appearing at 944 eV indicated the presence of CuO and polycrystalline Cu_2_O species in the sample [21,22,23]. The results indicate that CuO and Cu_2_O species may exist on the sample surface [24,25,26].

In Figure 8c, the O1s spectrum of the catalyst contains two peaks. The first peak is located in the range of 530–530.6 eV, which corresponds to lattice oxygen (O_α_). The second peak is located in the range of 531.1–531.6 eV and it represents the adsorption of hydroxyl or oxygen functional groups (O_β_) [14,23,27,28]. Similar to the results of Mn^3+^/Mn^4+^, the value of the Cu^2+^/Cu^+^ and the O_α_/O_β_ reduces after sulfur poisoning, and it increases after regeneration. The effect of the water washing regeneration is more obvious than the thermal regeneration. The XPS spectrum of S2p is also studied, displayed in Figure 8d. The peak appearing at 162 eV belongs to the low binding energy component [29,30]. The peak occurring at 163.5 eV is considered unbound sulfur, probably due to the physical adsorption of molecules and degradation by incident X-ray radiation [31]. The peak between 166 and 171 eV represents the presence of sulfur oxides [32], between 166 and 167 eV are sulfites, and between 169 and 171 eV are metal sulfates. From Table 2, it can be concluded that sulfur poisoning results in an increase in the amount of sulfate on the catalyst surface, and the water washing regeneration can reduce the content of sulfate; thus, the catalytic activity is recovered. In conclusion, the catalyst activity is related to Mn^3+^/Mn^4+^, Cu^2+^/Cu^+^, and O_α_/O_β_. Sulfur poisoning leads to the formation of sulfate and reduces Mn^3+^/Mn4^+^, Cu^2+^/Cu^+^, and O_α_/O_β_ on the catalyst surface; thus, the catalytic activity is reduced, but the activity could be recovered by the regeneration treatment, particularly the water washing regeneration. This is because the water washing regeneration reduces sulfite and sulfate, and returns the value of Mn^3+^/Mn^4+^, Cu^2+^/Cu^+^, and O_α_/O_β_ basically back to the level of the fresh catalyst.

## 4. Conclusions

In the current work, the effects of different factors and regeneration method on catalytic performance of the Mn–Cu/AC catalysts for SCR reaction are studied. The characterizations, including BET, XPS, XRD, SEM and FTIR, are performed to understand the physical structure and chemical properties of the catalysts and further uncover the underlying mechanisms. Results suggested that the 8 wt.% Mn_0.7_Cu_0.3_/AC catalyst, prepared by the incipient wetness impregnation method and calcined at 200 °C, has the highest NO conversion. Moreover, the NO conversion decreases with the increase of reaction space velocity, and the catalyst is poisoned and deactivated under the conditions of SO_2_. The activity of the sulfur poisoning catalyst could be recovered through thermal regeneration and water washing regeneration, and the effect of water washing regeneration is more remarkable. Based on characterization results, the water washing regeneration method could recover the specific surface area and total pore volume of the poisoning catalyst, destroy the C-S bond, reduce sulfite and sulfate on surface, and return the value of Mn^3+^/Mn^4+^, Cu^2+^/Cu^+^, and O_α_/O_β_ basically back to the level of the fresh catalyst; thereby, the catalytic activity is recovered after regeneration.

## Figures and Tables

**Table 1:** Specific surface area and pore size analysis.

Serial Number	Samples	Specific Area	Pore Volume	Pore Diameter
		m^2^/g	cm^3^/g	nm
a	AC	195.18	0.25	4.04
b	Fresh catalyst	207.36	0.26	3.99
c	After deNO_X_	172.04	0.23	3.77
d	Sulfur poisoning	81.45	0.17	3.78
e	Thermal regeneration	99.71	0.16	3.75
f	Washing regeneration	162.72	0.22	3.76

**Table 2:** XPS characterization results and surface element composition.

Sample	Mn^3+^	Mn^4+^	Mn^3+^/Mn^4+^	Cu^+^	Cu^2+^	Cu^2+^/Cu^+^	O_α_	O_β_	O_α_/O_β_	SO_3_^2−^	SO_4_^2−^	SO_3_^2−^/SO_4_^2−^
AC	-	-	-	-	-	-	530.2	531.3	1.3%	-	-	-
Fresh catalyst	641.2	642.7	2.6%	934.4	933.7	1.4%	530.2	531.1	1.8%	-	-	-
Sulfur poisoning	641.2	642.7	1.0%	934.7	933.8	0.6%	530.2	531.2	1.2%	166.03	169.14	0.2%
Thermal regeneration	641.3	642.7	1.2%	935.1	933.7	0.9%	530.6	531.5	1.0%	165.84	169.84	0.4%
Rinsing regeneration	641.2	642.7	2.6%	935.8	933.7	1.4%	530.5	531.5	1.6%	167.25	169.35	0.1%

## Data Availability

All experimental data required to repeat the analysis in this study are included in the article.
